# Congenital intrahepatic portosystemic venous shunt embolization: A two‐case experience

**DOI:** 10.1002/ccr3.2784

**Published:** 2020-03-05

**Authors:** Maria del Pilar Bayona Molano, Andres Krauthamer, Juan Carlos Barrera, Cibele Luna, Patricia Castillo, Adam Swersky, Shivank Bhatia

**Affiliations:** ^1^ University of Miami Miller School of Medicine/Jackson Memorial Hospital Miami FL USA; ^2^ Kent State University Kent OH USA; ^3^ Central University of Venezuela Caracas Venezuela

**Keywords:** congenital intrahepatic portosystemic shunts, congenital portal vein malformations, contrast‐enhanced computer tomography, encephalopathy, hepatic venography

## Abstract

Congenital intrahepatic portosystemic venous shunts are rare vascular malformations which are incidentally discovered on imaging or once hepatic encephalopathy becomes clinically apparent. Surgical ligation and endovascular embolization are potential treatments.

## Graphical abstract

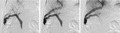

## 1 INTRODUCTION

Congenital intrahepatic portosystemic venous shunts are rare vascular malformations consisting of an abnormal connection between branches of the portal veins and hepatic veins. They often go undiagnosed and are incidentally discovered on imaging or once hepatic encephalopathy becomes clinically apparent. We present two cases of incidental congenital portosystemic shunts in noncirrhotic patients who went on to develop refractory encephalopathy without having prior history of liver disease. Both patients were successfully treated with embolization. Familiarity with the pathogenesis and imaging features may enable prompt diagnosis and help guide appropriate patient endovascular or surgical management.

Congenital intrahepatic portosystemic venous shunts (IPSVS) and extrahepatic portosystemic shunts make up the so called congenital portosystemic shunts (CPSS). Congenital portosystemic shunts are an important disorder in children and should be differentiated from metabolic deficiencies involving hyperammonemia or galactosemia.^1^ Intrahepatic shunts have a higher rate of spontaneous closure.^2^ In contrast, extrahepatic shunts almost never show spontaneous closure.^3^ Morgan and Superina classified the extrahepatic portosystemic shunts in two types considering the type I, a shunt that causes complete diversion of portal flow into the inferior vena cava with congenital absence of a portal vein. In addition, the splenic and mesenteric veins end up each one directly or in a common trunk into the vena cava. In type 2, the portal vein is intact, but some of the portal flow is diverted into the vena cava through a side‐to‐side extrahepatic communication.^1^, ^4^ Type II or partial shunts demonstrated a remaining degree of hepatic portal perfusion.^7^

Congenital intrahepatic portosystemic venous shunts (IPSVS) have a prevalence of 1 in 30 000 births and are caused by abnormal involution of the fetal vasculature. In these patients, there is a persistent communication between vitelline veins of the omphalomesenteric system and the sinus venosus due to a focal absence of sinusoid formations**.** These abnormalities include intrahepatic connections between branches of portal vein and hepatic veins.^1^ There are four morphological types of congenital IPSVS described in the literature: Type 1 is the most common and consists of a single vessel directly communicating the right portal vein with the inferior vena cava (IVC).^5^ Type II shunts involve one hepatic segment and consist of either a single communication or multiple communications between peripheral portal and hepatic venous branches.^3^ The third type of shunt is the least common and involves direct communication between peripheral portal and hepatic venous branches through an aneurysmal segment.^4^ The fourth type of shunt involves more than one hepatic segment and consists of multiple communicating peripheral portal and hepatic venous branches.^5^ Small intrahepatic shunts may resolve spontaneously within one year of age, but others should be closed with different surgical techniques.^6^

Kanasawa et al proposed a classification based on the correlation of the severity of portal hypoplasia (mild, moderate, and severe) with portal venous pressure, histopathological findings, postoperative portal venous flow, and liver regeneration.^7^ Interventional findings in Kanasagua's study demonstrated that performing a balloon‐occlusion test, the portal venous pressure was 9.7 ± 2.0 mm Hg before the test, 22.3 ± 9.5 mm Hg immediately after, and 19.3 ± 8.5 mm Hg, 15 minutes after shunt balloon occlusion.^7^

Congenital IPSVS can present with various symptoms and ultimately can lead to long‐term complications by permitting hepatic bypass of mesenteric venous blood return.^7^

Over time, unprocessed portal metabolites result in hyperammonemia and subsequent hepatic encephalopathy.^9^ Patients with shunts that go unrecognized may be misdiagnosed with psychiatric and/or neurologic disorders.^5^ Doppler ultrasound has been described as one of the most important diagnostic tools which help to calculate the portovenous shunt ratio by dividing the total blood flow volume in the shunt by that in the portal vein.^10^ Blood flow volumes are measured by multiplying the lumen area by the mean velocity at a given point.^4^, ^10^ Kudo et al demonstrated that shunt ratios of <24%‐30% do not cause liver encephalopathy, even in cirrhotic patients as opposed to patients of any age with shunts above 60% that should be corrected due to the risk of encephalopathy and liver dysfunction.^10^ Liver dysfunction is secondary to poor portal venous flow and lack of nutrition in the hepatic cells. The liver undergoes fatty degeneration and atrophy, but when the anomaly is corrected, fatty replacement disappears and liver size increases.^4^ Conservative medical therapy including restriction of protein and ingestion of lactulose, surgical ligation, and endovascular embolization has been described as potential treatment alternatives for these shunts. Tanoue et al reported 10 patients with symptomatic intrahepatic portovenous shunts effectively treated by transvenous embolization using three different approaches: transileocolic, percutaneous transhepatic, or retrograde transcaval via perihepatic veins with mild complication rates.^11^

If the medical or operative treatment fails, liver transplantation is the only therapeutic option being cautious with major hemodynamic changes that can occur in the intraoperative period. Resection is the treatment of choice in patients when liver tumors are associated with extrahepatic portosystemic shunting.

We describe two cases of congenital intrahepatic portosystemic shunts, discovered incidentally in noncirrhotic adult patients who develop refractory encephalopathy without having history of liver disease or prior surgical intervention. The two patients were successfully treated using transcatheter embolization with clinical improvement. Endovascular treatment offers a safe alternative of therapy after evaluation of anatomical and pathophysiological characteristics of the anomaly.

### 1.1 Patient 1

**Figure 1:**
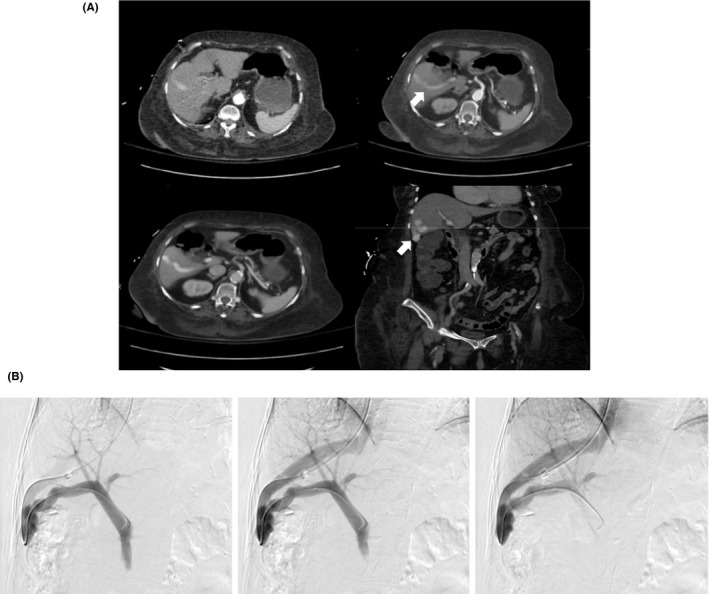
A 75‐year‐old woman with congenital intrahepatic portosystemic venous shunt. A, Pre‐embolization axial and coronal images from a contrast‐enhanced CT of the abdomen and pelvis which demonstrates an enlarged right posterior portal vein and right hepatic vein with significant enhancement in the arterial phase. There is vascular tortuosity at the level of the shunt (white arrow). B, Portal venography with fluoroscopic DSA obtained with transjugular access and catheter positioning across the intrahepatic portosystemic shunt into the main portal vein. This venogram confirmed the communication between the right hepatic and right posterior portal veins

A 75‐year‐old woman with no significant history of liver disease or neurologic disorder was evaluated for altered mental status. Her initial ammonia level was 33 mmol/L (normal range 11‐32 mmol/L), and liver function tests (LFTs) were within normal range. A CT of the abdomen was performed which demonstrated an intrahepatic shunt between the right posterior portal vein and the right hepatic vein (Figure 1A) as well as enlargement of the right hepatic vein (type II IPSVS). No additional vascular abnormalities were identified. Given the lack of prior surgery or biopsy, the etiology of this shunt was deemed to be congenital in nature.

After consultation with the interventional radiology service, angiography with endovascular embolization of the shunt was planned. The right hepatic vein was catheterized from a right internal jugular venous approach, and a 4‐French angled glide catheter (Terumo Corporation) was manipulated through the shunt into the main portal vein. Prior to embolization, a temporary balloon‐occlusion test of the shunt was performed using a 12‐mm Berenstein occlusion balloon catheter (Boston Scientific). The test did show a portal pressure of 9 mm Hg before and 16 mm Hg after balloon occlusion, and this lasts remaining unchanged after several minutes. The findings were interpreted as no significant hemodynamic changes or risk of portal hypertension that would preclude shunt occlusion. Therefore, the shunt was then successfully embolized with Penumbra POD coils (Penumbra). Due to the high flow nature of the shunt, it was necessary to use coils that were approximately 50% oversized for the targeted vessel diameter to prevent potential coil migration and nontarget embolization. Following POD embolization, an Amplatzer Vascular Plug II (St. Jude Medical) was deployed as a safety measure to further avoid nontarget coil migration into the right atrium and pulmonary vasculature.

**Figure 2:**
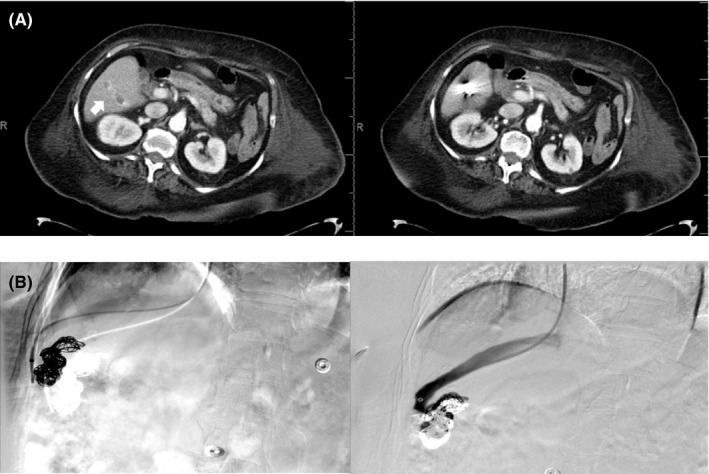
A 75‐year‐old woman with congenital intrahepatic portosystemic venous shunt. A, Postembolization axial images from a contrast‐enhanced CT of the abdomen and pelvis showing embolic material at the periphery of the liver and no further opacification of the right portal to hepatic vein shunt. Of note, there is thrombosis of the shunt at the periphery of some posterior segmental branches of the right portal vein (white arrow). B, Catheterization of the right hepatic vein with subsequent venogram and digital subtraction images demonstrated opacification of the vessel without visualization of the preexisting portosystemic shunt. Multiple coils are visualized at the proximal vein consistent with postembolization material. No complications were identified

Following embolization, a contrast‐enhanced CT of the abdomen and pelvis was performed which demonstrated successful exclusion of the venous‐venous malformation with thrombosis of the posterior segment branch of the right portal vein (Figure 2). Follow‐up at 6 and 12 months later yielded a normalized ammonia level and over time, improvement and objective resolution of the patient's hepatic encephalopathy. Abdominal color Doppler ultrasound did not show recurrent shunt recanalization.

### 1.2 Patient 2

**Figure 3:**
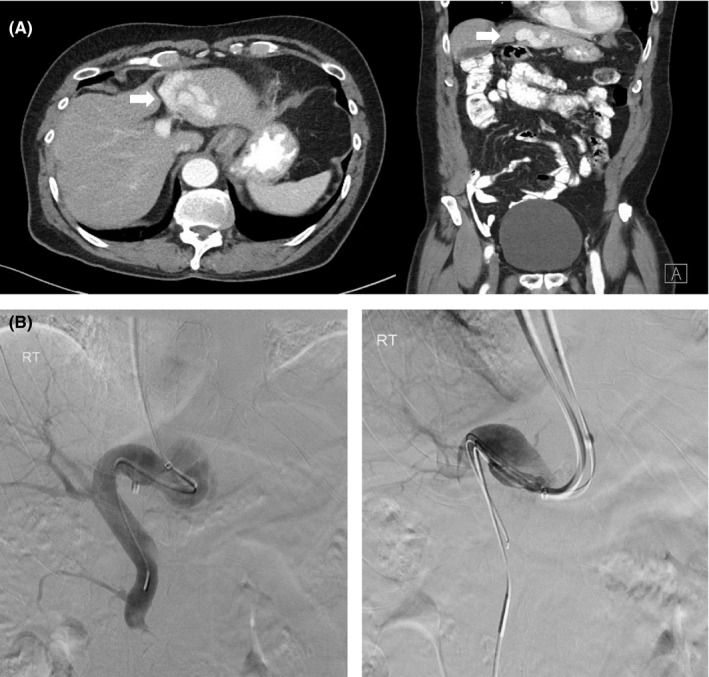
A 72‐year‐old man with congenital intrahepatic portosystemic venous shunt. A, Axial and coronal images from the pre‐embolization contrast‐enhanced CT of the abdomen and pelvis which demonstrates the abnormal enlarged intrahepatic veins corresponding to left portal vein to left hepatic vein shunt (white arrow). B, Portal venography with fluoroscopic DSA obtained using transjugular access through the left hepatic vein into the main portal vein across the spontaneous shunt

**Figure 4:**
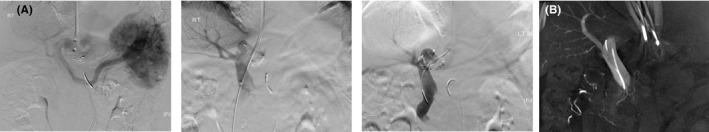
A 72‐year‐old man with congenital intrahepatic portosystemic venous shunt. A, Fluoroscopic DSA portal venogram. B, Coronal MIP from cone‐beam CT of postembolization portal venogram which demonstrates successful occlusion of the intrahepatic portosystemic (left portal vein to left hepatic vein) shunt

A 72‐year‐old man with no significant history of liver disease, neurologic disorder, or prior relevant surgical intervention presented with the acute onset of lower extremity weakness and confusion. A full neurological workup was performed which included a normal electro encephalogram (EEG) and an unremarkable MRI of the brain. An ammonia level of 145 mmol/L (normal range 11‐32 mmol/L) was measured which suggested hepatic encephalopathy as the cause of the patient's altered mental status. A contrast‐enhanced CT of the abdomen and pelvis was then performed which demonstrated an intrahepatic portosystemic shunt between the left portal vein and the left hepatic vein with multiple venous connections (Figure 3A). After consultation with the interventional radiology service, angiography with endovascular embolization of the shunt was planned and began with transhepatic access of the right portal vein from a transjugular approach using a Colapinto needle; however, the right portal vein was small in caliber due to retrograde flow across the existing portosystemic shunt. Left hepatic to left portal venous access was subsequently obtained utilizing a transjugular approach via manipulation of the catheter across the left hepatic vein. The main portal vein was then catheterized with an MPA catheter followed by a direct portal venogram which demonstrated three separate communicating branches. The pressure of the main portal vein was measured before the procedure and corresponds to 6 mm Hg before balloon inflation. Another transjugular access was obtained to catheterize the second more prominent branch. A balloon‐occlusion test was then performed simultaneously occluding the two more prominent shunts as a safety measure to mimic any potential hemodynamic changes following embolization obtaining a sustained increase in the main portal vein pressure up to 14 mm Hg interpreted as low risk for embolization. Next, the three communicating branches were embolized using Amplatzer Vascular Plugs II (St. Jude Medical). Each plug was oversized by approximately 30% relative to the shunt caliber. There were no complications following completion of the procedure. A postembolization triple‐phase contrast‐enhanced CT of the abdomen and pelvis was performed which demonstrated successful occlusion of the shunt (Figure 4). Shortly following embolization, the patient's ammonia level returned to normal and his encephalopathy resolved. Follow‐up 6 months later, a liver Doppler ultrasound showed no recurrence of shunts and the ammonia levels remained within normal limits.

## 2 DISCUSSION

Intrahepatic portosystemic shunts are often incidentally detected on imaging or diagnosed after symptoms of hepatic encephalopathy develop. The incidence has been reported as 1/30 000. They have been associated with other types of anomalies such as cardiovascular, hepatobiliary, urogenital, and gastrointestinal among others. In addition, complications like portopulmonary hypertension, reported in 13%‐66% in children,^12^ hepatic encephalopathy, and hepatopulmonary syndrome are the most prominent manifestations caused by long‐term shunting.^8^ Congenital intrahepatic portosystemic venous shunt may close spontaneously within the first 2 years of life or may remain asymptomatic and undetected for several years.^13^ Alternatively, some shunts present in infancy and manifest in neonatal hyperammonemia which has been explained by some authors due to some precipitating factor like constipation.^5^ When chronic shunting persists into adulthood, patients most often present with encephalopathy; however, pulmonary arterial hypertension and/or heart failure have both been described in the literature as alternative presentations.^14^ Because of the complications of long‐standing portosystemic shunting, endovascular embolization should be considered, making them potentially reversible by closing the shunt.^5^, ^15^ Guerin et al reported two major challenges of endovascular embolization: The first one related with portal hypertension after the shunt is closed, given the noncompliant portal venous system to the restoration of full flow, and the second one related with the possibility of coils or plug migration in certain focal narrow shunts.^12^ Technical strategies to be used are the balloon‐assisted portography for better evaluation of the anatomy and continuous monitorization of the portal venous pressure to avoid potential portal hypertension. The use of ethanol, coils, and Amplatzer Vascular Plugs have all been reported as effective means of shunt closure.^13^ Takenaga et al described one case of multiple intrahepatic portosystemic shunts successfully treated with transhepatic embolization.^16^

Multiple imaging modalities are useful for diagnosis of an intrahepatic portosystemic venous shunt including color Doppler ultrasonography, contrast‐enhanced CT, MRI or conventional catheter‐directed angiography. The imaging finding most consistent with diagnosis of a portosystemic venous shunt is visualization of a direct communication between portal and hepatic veins; however, this is not always demonstrated on imaging. Secondary findings on imaging suggestive of a shunt include abnormal blood pooling from a dilated portal branch with early visualization of the hepatic venous system.^17^ In our cases, contrast‐enhanced CT detected the abnormal direct communication between the portal vein and hepatic vein. We believe both cases were congenital in nature given no other potential etiologies for these patients.

Multiple challenges can be present during the embolization including diminutive portal vein in large portosystemic shunts, difficulty to determine the anatomy especially in types II or IV if multiple venous communications can be present or large veins with potential risk of coils or plug migration. Optimal oversize of the plugs or coils is recommended to avoid any potential undesired migration to the systemic circulation. Patient follow‐up is mandatory to evaluate for the ammonia levels and to determine whether further embolization is required.

## 3 CONCLUSION

Intrahepatic portosystemic venous shunts are rare hepatic vascular malformations that often remain asymptomatic and are discovered incidentally on imaging. Alternatively, these patients may present with hyperammonemia and encephalopathy among other symptoms, and with proper endovascular management, this detrimental sequela is potentially reversible. Technical challenges during endovascular treatment could be overpassed with detailed evaluation of the anatomy as well as hemodynamic changes including identifying multiple potential venous communications, measuring portal pressure, and selection of the more appropriate embolization material.

## CONFLICT OF INTEREST

None declared.

## AUTHOR CONTRIBUTION

MPBM: served as the primary author; was responsible for accuracy and integrity of the case report; and involved in the conception of the article, performed the interventional radiology procedures of each case, collected the radiologic imaging studies, reviewed the manuscript drafts, and approved and modified all versions of the manuscript. AK: did the literature review and wrote the background of the manuscript. JCB: served as the coauthor; did the modifications and corrections suggested by the reviewers; complemented the background, results, and description of the cases; and additionally corrected and supplemented the references of the last manuscript. CL: served as the coauthor and helped to write the first draft of the manuscript. PC: served as the coauthor, and interpreted and read the diagnostic CT images. AS: is the corresponding author; involved in design and writing of drafts including all the manuscript sections; uploaded the images; collected the cases; and submitted the case report every time when needed. SB: served as the coauthor; reviewed the drafts; helped in the interpretation of interventional radiology images; and helped in maintaining the accuracy of all aspects of the manuscript.

## References

[1] Gallego C , Miralles M , Marin C , Muyor P , Gonzalez G , Garcia‐ HE . Congenital hepatic shunts. Radiographics. 2004;24:755‐772. 15143226 10.1148/rg.243035046

[2] Sokollik C , Bandsma RHJ , Gana JC , van den Heuvel M , Ling SC . Congenital portosystemic shunt: characterization of a multisystem disease. J Pediatr Gastroenterol Nutr. 2013;56:675‐681. 23412540 10.1097/MPG.0b013e31828b3750

[3] Weigert A , Bierwolf J , Reutter H , et al. Congenital intrahepatic portocaval shunts and hypoglycemia due to secondary hyperinsulinism: a case report and review of the literature. J Med Case Rep. 2018;12:336. 30415638 10.1186/s13256-018-1881-y PMC6231275

[4] Morgan G , Superina R . Congenital absence of portal vein: two cases and a proposed classification system for portasystemic vascular anomalies. J Pediatr Surg. 1994;29(9):1239‐1241. 7807356 10.1016/0022-3468(94)90812-5

[5] Palvanov A , Marder R , Siegel D . Asymptomatic intrahepatic portosystemic venous shunt: to treat or not to treat? Int J Angiol. 2016;25(3):193‐198. 27574389 10.1055/s-0035-1564658 PMC5001872

[6] Bernard O , Franchi‐Abella S , Branchereau S , Parient D , Gautier F , Jacquemin E . Congenital portosystemic shunts in children: recognition, evaluation, and management. Semin Liver Dis. 2012;32(4):273‐287. 23397528 10.1055/s-0032-1329896

[7] Kanazawa H , Nosaka S , Miyazaki O , et al. The classification based on intrahepatic portal system for congenital portosystemic shunts. J Pediatr Surg. 2015;50:688‐695. 25840084 10.1016/j.jpedsurg.2015.01.009

[8] Papamichail M , Pizanias M , Heaton N . Congenital portosystemic venous shunt. Eur J Pediatr. 2018;177:285‐294. 29243189 10.1007/s00431-017-3058-x PMC5816775

[9] Gong Y , Zhu H , Chen J , et al. Congenital portosystemic shunts with and without gastrointestinal bleeding: case series. Pediatr Radiol. 2015;45(13):1964‐1971. 26209117 10.1007/s00247-015-3417-6

[10] Kudo M , Tomita S , Tochio H , Minowa K , Todo A . Intrahepatic portosystemic venous shunt: diagnosis by color Doppler imaging. Am J Gastroenterol. 1993;88:723‐729. 8480738

[11] Tanoue S , Kiyosue H , Komatsu E , Hori Y , Maeda T , Mori H . Symptomatic intrahepatic portosystemic venous shunt: embolization with an alternative approach. AJR Am Roentgenol. 2003;181(1):71‐78. 10.2214/ajr.181.1.1810071 12818832

[12] Guerin F , Blanc T , Gauthier F , et al. Congenital portosystemic vascular malformations. Semin Pediatr Surg. 2012;21:233‐244. 22800976 10.1053/j.sempedsurg.2012.05.006

[13] Brader R , Kim K . Transhepatic embolization of a congenital intrahepatic portosystemic shunt for the treatment of hepatic encephalopathy in a noncirrhotic patient using Amplatzer vascular plug device. Radiol Case Rep. 2017;12(2):318‐322. 28491179 10.1016/j.radcr.2016.12.006 PMC5417625

[14] Tsitouridis I , Sotiriadis C , Michaelides M , Dimarelos V , Tsitour‐idis K , Stratilati S . Intrahepatic portosystemic venous shunts: radiological evaluation. Diagn Interv Radiol. 2009;15(3):182‐187. 19728264

[15] Lee Y‐J , Shin BS , Lee IH , et al. Intrahepatic portosystemic venous shunt: successful embolization using the Amplatzer Vascular Plug II. Korean J Radiol. 2012;13(6):827–831. 23118586 10.3348/kjr.2012.13.6.827 PMC3484308

[16] Takenaga S , Narita K , Matsui Y , Fukuda K . Hepatic encephalophalopathy due to congenital Multiple Intrahepatic Portosystemic Venous shunts successfully treated by percutaneous transhepatic obliteration. Case Rep Gastroenterol. 2016;10:701‐705. 27990104 10.1159/000452204 PMC5156888

[17] Pocha C , Maliakkal B . Spontaneous intrahepatic portal‐systemic venous shunt in the adult: case report and review of the literature. Dig Dis Sci. 2004;49(7–8):1201‐1206. 15387347 10.1023/b:ddas.0000037813.24605.d5

